# Unveiling the Inhibitory Potentials of Peptidomimetic Azanitriles and Pyridyl Esters towards SARS-CoV-2 Main Protease: A Molecular Modelling Investigation

**DOI:** 10.3390/molecules28062641

**Published:** 2023-03-14

**Authors:** Aganze G. Mushebenge, Samuel C. Ugbaja, Sphamandla E. Mtambo, Thandokuhle Ntombela, Joy I. Metu, Oludotun Babayemi, Joy I. Chima, Patrick Appiah-Kubi, Adeshina I. Odugbemi, Mthobisi L. Ntuli, Rene Khan, Hezekiel M. Kumalo

**Affiliations:** 1 Drug Research and Innovation Unit, Discipline of Medical Biochemistry, School of Laboratory Medicine and Medical Science, University of KwaZulu-Natal, Durban 4000, South Africa; aganzedar@gmail.com (A.G.M.); sphamtambo@gmail.com (S.E.M.); joieugbaja@gmail.com (J.I.C.); appiahpat@gmail.com (P.A.-K.); myburgr@ukzn.ac.za (R.K.); 2 Catalysis and Peptide Research Unit, School of Pharmaceutical Sciences, University of KwaZulu-Natal, Durban 4000, South Africa; ntombelathandokuhle@gmail.com; 3 National Institute for Nigerian Languages, Aba 453106, Nigeria; joyimetu@yahoo.com; 4 Cloneshouse Nigeria, 6th Floor, Left Wing, NICON Plaza, Plot 242, Muhammadu Buhari Way, Central Business District, Abuja 900103, Nigeria; oludotunbabayemi@cloneshouse.com; 5 South African National Bioinformatics Institute, Faculty of Natural Sciences, University of the Western Cape, Cape Town 7535, South Africa; adeshina.odugbemi@gmail.com; 6 Department of Mathematics, Faculty of Applied Science, Durban University of Technology, Durban 4000, South Africa; mthobisin2@dut.ac.za

**Keywords:** SARS-CoV-2 main protease, ADME, binding free energy, molecular dynamics simulations

## Abstract

The severe acute respiratory syndrome coronavirus-2 (SARS-CoV-2) is responsible for COVID-19, which was declared a global pandemic in March 2020 by the World Health Organization (WHO). Since SARS-CoV-2 main protease plays an essential role in the virus’s life cycle, the design of small drug molecules with lower molecular weight has been a promising development targeting its inhibition. Herein, we evaluated the novel peptidomimetic azatripeptide and azatetrapeptide nitriles against SARS-CoV-2 main protease. We employed molecular dynamics (MD) simulations to elucidate the selected compounds’ binding free energy profiles against SARS-CoV-2 and further unveil the residues responsible for the drug-binding properties. Compound **8** exhibited the highest binding free energy of −49.37 ± 0.15 kcal/mol, followed by compound **7** (−39.83 ± 0.19 kcal/mol), while compound **17** showed the lowest binding free energy (−23.54 ± 0.19 kcal/mol). In addition, the absorption, distribution, metabolism, and excretion (ADME) assessment was performed and revealed that only compound **17** met the drug-likeness parameters and exhibited high pharmacokinetics to inhibit CYP1A2, CYP2C19, and CYP2C9 with better absorption potential and blood-brain barrier permeability (BBB) index. The additional intermolecular evaluations suggested compound **8** as a promising drug candidate for inhibiting SARS-CoV-2 Mpro. The substitution of isopropane in compound **7** with an aromatic benzene ring in compound **8** significantly enhanced the drug’s ability to bind better at the active site of the SARS-CoV-2 Mpro.

## 1. Introduction

Recently, there has been increased mortality arising from the upsurge of the unexpected severe respiratory syndrome coronavirus2 (SARS-CoV-2), responsible for the COVID-19 pandemic in December 2019 [1,2]. Prior to SARS-CoV-2, two different coronaviruses caused large-scale disease outbreaks. Severe acute respiratory syndrome coronavirus (SARS-CoV) was the first to be reported in 2002, followed by the Middle East respiratory coronavirus MERS-CoV in 2012 [3,4]. Sequel to the first reported incidence of (COVID-19) in Wuhan, China, the World Health Organization (WHO) in March 2020 declared COVID-19 a global pandemic [5]. COVID-19 has an unprecedented rate of infection and transmission, with over 280 million cases and over 5.7 million reported deaths globally as of February 2022 [5]. Following a WHO epidemiological update as of 22 June 2021, four different SARS-CoV-2 variants of concern (VOC) have been identified. Alpha (B.1.1.7) was the first VOC reported in December 2020 in the United Kingdom. The second identified VOC was Beta (B.1.351), first reported in South Africa in December 2020. Gamma (P.1) was the third identified VOC, reported first in Brazil in January 2021. The fourth VOC is Delta (B.1.617.2) which was first identified in India in December 2020 [5,6]. A recently updated World Health Organization epidemiology report states that over 200 countries globally have recorded SARS-CoV-2 variants of concern (VOC). Omicron is the most recent VOC, first reported in November 2021 and has already been reported in 171 countries as of 21 January 2022. COVID-19 is a great threat to persons of all ages, especially those above 60 years, and also persons with underlying medical health conditions are at higher risk [5,6].

**Figure 1:**
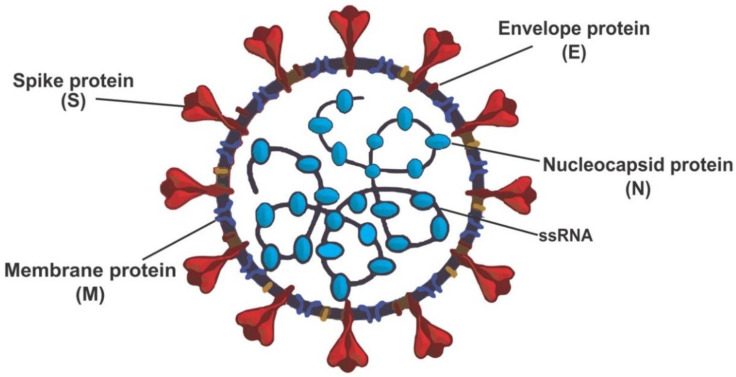
Structure of severe acute respiratory coronavirus-2 (SARS-CoV-2) redrew from open access journals [7,8].

Both SARS-CoV and SARS-CoV-2 genomes encode four different structural proteins, which help build the spike and globular shapes. These structural proteins (Figure 1) are spike protein (S), an envelope protein (E), membrane protein (M), and nucleocapsid protein (N). SARS-CoV and SARS-CoV-2 viruses enter the host cells by binding to the angiotensin-converting enzyme-2 (ACE2) using the spike protein [7].

SARS-CoV-2 is mainly transmitted through respiratory droplets and aerosols from coughs or sneezes, which enter the host’s nose, eyes, or mouth. When the SARS-CoV-2 enters the host, it attaches to multiple different types of cells in the respiratory tract and replicates the same as SARS-CoV [9]. In addition to binding at the lower respiratory tract like SARS-CoV, SARS-CoV-2 binds to the upper respiratory tract, enhancing the rapid spread to other humans [10]. Understanding SARS-CoV-2 modes of binding and the amino acid residues responsible for its attachment to the host is also essential in drug design and the future development of vaccines against the virus. Given the severity of COVID-19, its impact on public life in lockdown mode, and the global economic ramifications, there is an unmet medical need to create clinically effective SARS-CoV-2-specific drugs [1]. Recently Breidenbach et al. (2021) employed quantitative high-throughput screening (HTS) and a comparative study to the current standard assay in the discovery of novel SARS-CoV-2 main protease inhibitors [1]. The authors further applied structure-based drug design, synthesis, and biochemical characterisation of highly potent inhibitors for SARS-CoV-2 main protease inhibitors. Over the years, efforts of researchers in developing antiviral compounds in the fight against COVID-19 have proven the importance of Mpro as the main target [11]. The structure of Mpro, as revealed by X-ray crystallography, unveiled the importance of the catalytic residues (Glu166, Cys141, and His41) in the binding and subsequent inhibition mechanism activities of the enzyme [11]. Lately, studies have explored in vivo and in silico studies in the fight against the SAR-CoV-2 main protease. Recently, the therapeutic potentials of heterocyclic compounds have been explored in treating diseases like cancer and SARS-CoV-2. Fluorescence resonance energy transfer (FRET) assay unveiled the inhibition potential of benzothiophenemethyl with 5-iodo isatin (IC50 value of 0.95 μM) to SAR-CoV-2 main protease [12,13]. Another study also reported higher inhibitory potentials of benzothiophenemethyl with 5-carboxamide and 5-sulfonamide (other analogues of isatin) relative to 5-iodo isatin [14,15]. Through drug repurposing, chloroquine 10, an anti-malarial drug, yielded a high IC50 value of 8.8 μM through MTT assay in the inhibition of SAR-CoV-2 Mpro. Another study further substantiated this finding, which reported that chloroquine 10 could act as a prophylaxis against the COVID-19 pandemic when treated with the stipulated concentration and time after the viral attack [13,16]. 

**Figure 2:**
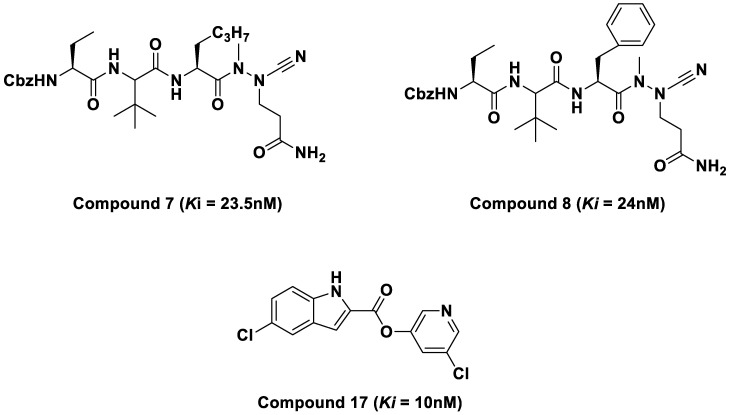
2D structures of azatripeptide and azatetrapeptide nitrile compounds.

Furthermore, in silico studies also revealed the inhibitory potentials of purine and pyrimidine compounds against the SARS-CoV-2 Mpro as reported in the literature [17,18]. This study investigated three novel SARS-CoV-2 inhibitors and unveiled the molecular interactions responsible for their potency against SARS-CoV-2 main protease (Mpro). The compounds that exhibited high inhibitory constant values were azanitrile compounds **7** and **8** (Ki = 23.5 nM and 24 nM), and pyridyl ester compound **17** (Ki = 10 nM) [1]. These compounds (Figure 2) were selected for molecular dynamic (MD) simulations and post-MD analysis. This study aimed to provide theoretical insight into the inhibition efficiency of the selected compounds against SARS-CoV-2 Mpro at an atomic level. Furthermore, the compounds were evaluated for drug-likeness properties using the SwissADME web server.

## 2. Results and Discussions

**Figure 3:**
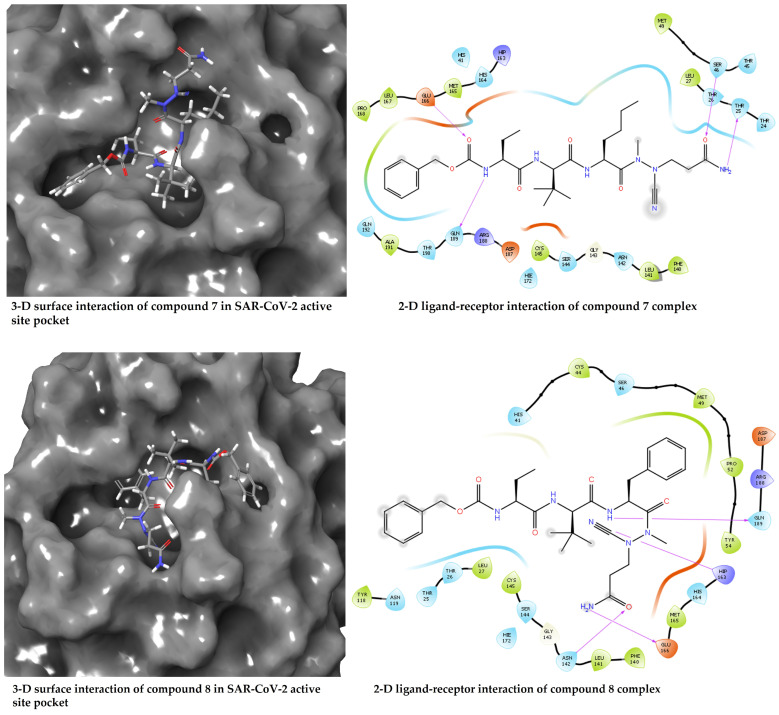

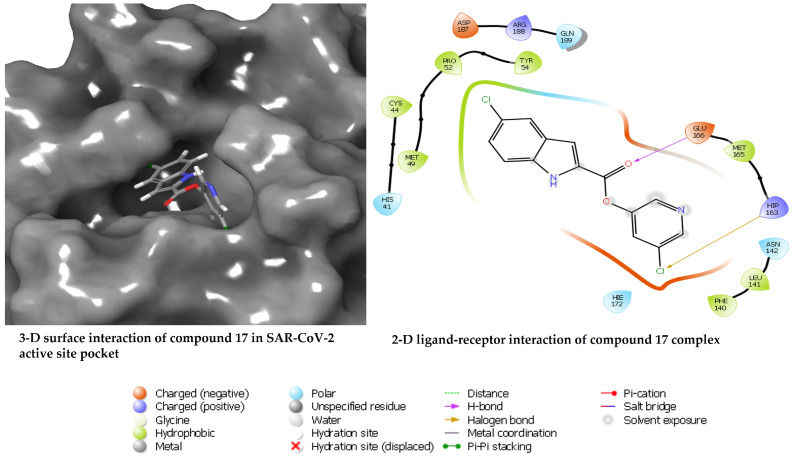
Diagrams of the binding of compounds **7**, **8**, and **17** at the binding site of SARS-CoV-2 Mpro.

The docked compounds **7**, **8,** and **17** showed the docking scores −6.56, −5.71, and −2.68 kcal/mol, respectively, suggesting compound **7** has better interaction with the receptor’s binding site when compared with compounds **8** and **17**. Given the structural similarity of compounds **7** and **8**, both docking scores are closer (−6.56 and −5.71 kcal/mol) than the distant compound **17**, with the least stable interaction based on the docking score. A closer look at the protein–ligand interaction suggests that the degree of hydrogen bonding interactions influences the differential binding observed among the docked complexes. Hydrogen bond interactions with GLU166 and GLN189 appear to be generally needed for a stable ligand–receptor interaction (Figure 3). This is true for all the examined ligands, including the redocked co-crystallised ligand, except for compound **17**, which has just one H-bond interaction with GLU166. Hydrogen bond interaction with THR25 and SER46 appears crucial for the stronger binding of compound **7**. It was observed that the binding affinity drops in compound **8** when these interactions disappear, probably due to hydrophobic interaction with MET49 and MET165 and the involvement of the –C≡N in hydrogen bond interaction. Moreover, the hydrogen bond interactions are well dispersed around compound **7**, which could afford more stability than compound **8**, which appears to be a bit skewed to a region of the ligand. Furthermore, the MD simulations were performed to unveil further the interaction of compounds **7**, **8,** and **17** when bound with the SAR-CoV2 Mpro.

### 2.1. MMGBSA Binding Free Energy Calculation

The SARS-CoV-2 main protease is an important drug target extensively investigated on the verge of deterring its role in the proliferation and maturation of new virions. Inhibition of SARS-CoV-2 main protease is crucial to prevent the assembling and maturation of new virions. Herein, three experimentally evaluated compounds were selected to theoretically investigate their binding affinities and further provide information pertinent to the underlying activity at an atomistic level. MD simulations were performed, and post-MD tractories were analysed using various metrics. As the primary focus of the study, the binding free energy calculations were performed for SARS-CoV-2 Mpro with compounds **7**, **8**, and **17** complexes employing the MMGBSA method on the stable MD trajectories. The total binding free energy (ΔG_bind_) results and other energy contributions for SARS-CoV-2 Mpro with compounds **7**, **8**, and **17** complexes are provided in Table 1. Compound **8** complex displayed the highest total binding energy of −49.37 ± 0.15 kcal/mol, followed by compound **7** with −39.83 ± 0.19 kcal/mol, and −23.54 ± 0.19 kcal/mol for compound **17**.

Furthermore, compound **8** exhibited the highest van der Waals (ΔG_vdw_), solvent, non-polar (ΔG_nonpol_), gas (ΔG_gas_), and electrostatic (ΔG_ele_) energies. The contributions of van der Waals (ΔG_vdw_) and electrostatic (ΔG_ele_) contributed significantly to the observed highest binding free energy in compound **8**. Also, from a structural point of view, the isopropane substitution with an aromatic benzene moiety suggests being crucial in the observed better binding potential of compound **8**.

The selected compounds’ pharmacokinetics profile, physiochemical, and drug-likeness were assessed using the SwissADME web server [19,20]. Table 2 showed that compounds **7** and **8** with molecular weight above 500 g/mol have more than five rotatable hydrogens, more than 5 H-bonds acceptors, and donors with no blood–brain barrier permeation. Conversely, compound **17**, with a molecular weight of 307 g/mol, have less than five rotatable hydrogen bonds, less than five H-bond acceptors, and donors exhibited good blood–brain barrier permeation potentials. 

Further analysis of the post-molecular dynamic simulations was carried out to thoroughly investigate and unveil the inhibitory potentials of these selected peptidomimetic azanitriles and pyridyl esters towards SARS-CoV-2 main protease at the intermolecular and interatomic levels. The following post-molecular dynamic simulations analyses were carried out, including root mean square fluctuation (RMSF), H-bond analysis, root mean square deviation (RMSD), a radius of gyration (RoG), solvent accessible surface area (SASA), principal component analysis (PCA), and binding free energy analysis.

### 2.2. Root Mean Square Deviation 

**Figure 4:**
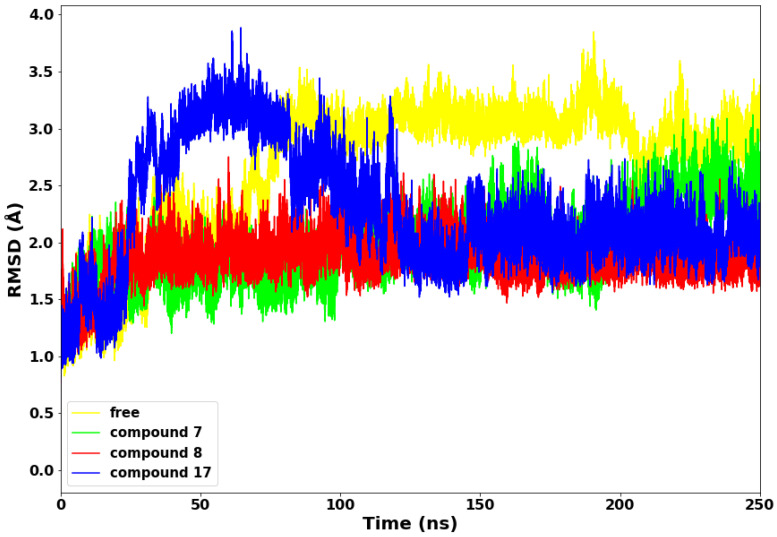
Root Mean Square Deviation (RMSD) plot of the a-C backbone of the protein with the selected compounds.

We analysed the stability of the different conformations of the SARS-CoV-2 main protease with compounds **7**, **8,** and **17**, and the apo aimed at comparing these biomolecular compounds’ structure and dynamic behaviour while calculating the root mean square deviation (RMSD) of backbone C-α atoms. The result further unveils the SARS-CoV-2 conformations throughout the molecular dynamic simulation run and the RMSD evolutionary trend of the protease. The RMSD evaluation for the four systems (SARS-CoV-2 apo and in complex with compounds **7**, **8**, and **17**) is presented in Figure 4. 

Compounds **7** and **8** maintained lower stable RMSD values throughout the 250 ns simulations run relative to the apo and compound **17**. The exhibited lower and stable conformations could be due to higher molecular weights of both compounds (**7** and **8**) and depicts decreased mobility and structural stability. Conversely, compound **17** displayed sudden higher conformations from 28 ns–110 ns of the simulations run. The higher deviations of about 3.8 Å are expected due to the lower molecular weight of compound **17** and the probable adjustment of the drug molecules to bind with the protein properly. The four systems converged from 127 ns to the end of the simulation run, with compounds **7**, **8**, and **17** depicting lower RMSD values.

### 2.3. Radius of Gyration 

**Figure 5:**
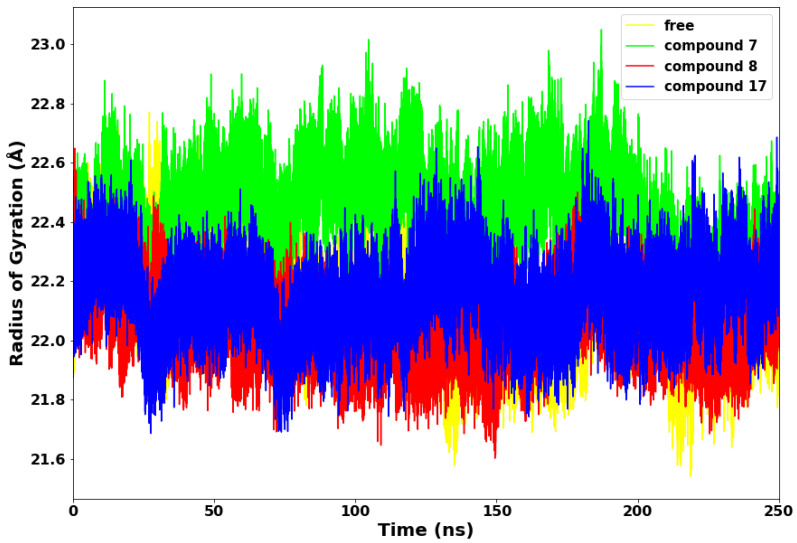
Radius of Gyration (RoG) plot.

The radius of gyration (RoG) is an essential technique for determining the compactness of protein structure. The RoG of the SARS-CoV-2 main protease with compounds **7**, **8**, **17**, and the apo is depicted in Figure 5. Compounds **8** and **17** maintained a lower radius of gyration, which could be due to decreased flexibility of backbone atoms, an indication of rigid structural stability. Subsequently, compound **7** displayed higher RoG with the highest peak at 23 Å, which is consistent with the fluctuations observed in the RMSF plot and suggests poor folding and less compactness in the complex. This observation indicates that compound **7** is relatively less stable in complex with the SARS-CoV-2 Mpro.

### 2.4. Solvent Accessible Surface Area

**Figure 6:**
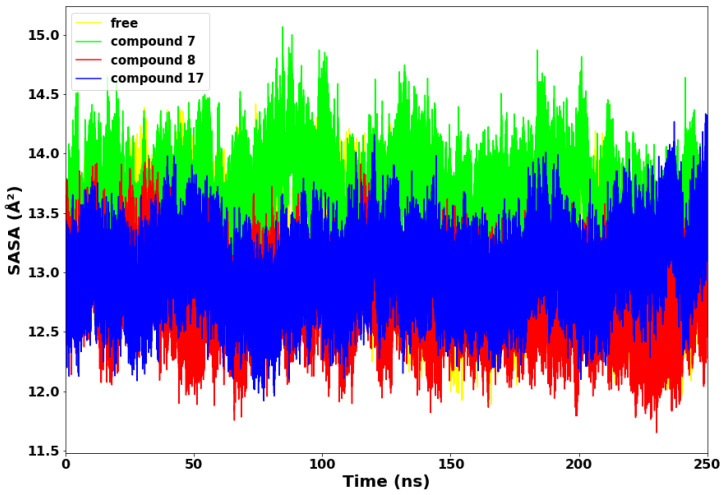
Solvent accessible surface area (SASA).

The surface properties of the SARS-CoV-2 Mpro and other proteins are mainly evaluated and determined by the interactions between proteins and ligands. It is imperative to understand the dynamisms of structural deviations and how it affects the solvent-accessible surface area (SASA) [21,22]. Surface accessible surface area measures the surface area of the protein that interacts with solvents. We further employed SASA to evaluate the degree of compactness of the SARS-CoV-2 main protease with compounds **7**, **8**, and **17**, as depicted in Figure 6. 

Compounds **8** and **17** maintained lower values of SASA, suggesting that both systems exhibited structural stability throughout the 250 ns simulations time. Compound **8** displayed the lowest SASA value of 11.6 Å^2^ at 235 ns. Conversely, compound **7** exhibited higher values of SASA from the beginning to the end of the simulation time. This suggests that the compound **7** complex allowed more of the protein to be exposed to water molecules. Therefore, compound **7** is thermodynamically unstable and less compact, while compound **8** is the most thermodynamically stable. 

### 2.5. Hydrogen Bond Network Profile

**Figure 7:**
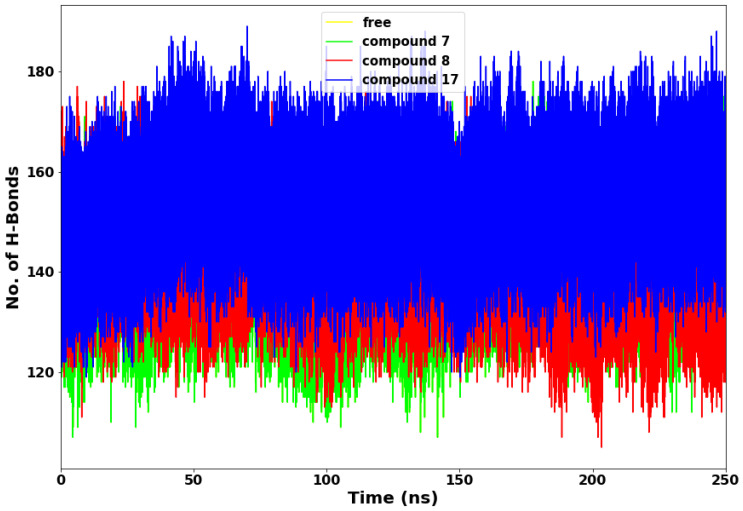
Hydrogen bond interaction plot.

Hydrogen bond evaluation is another important method for determining a protein’s stability [23]. The more intramolecular hydrogen bonds established by the active site residues with the ligand, the more rigid and compact the protein structure [24]. The plot of hydrogen bond in Figure 7 shows that compound **17** consistently maintained very high hydrogen bond interactions with the SARS-CoV-2 Mpro throughout the simulations compared to compounds **7** and **8**. This suggests that the drug molecule (compound **17**) has stronger hydrogen bonding with the protein’s amino acid residues than compounds **7** and **8**. The relatively lower hydrogen bond displayed by compounds **7** and **8** suggests less rigidity and unstable interactions between the compounds and SARS-CoV-2 Mpro (Table 3).

Furthermore, the hydrogen bond percentage (%) occupancy and the average distance (Å) between the compounds **7**, **8**, and **17** and the active site residues were monitored (Table 1) during the 250-ns simulations. The primary residues that maintain hydrogen bonds between protease and these compounds are His 41, Asn 142, Gly 143, Cys 145, His 164, and Glu 166. Therefore, a compound’s effectiveness is affected by its interaction with these primary residues. In compound **8**, the protease residues Gly 143 and His 164 form hydrogen bonds with a percentage occupancy of 46.96% and 63.41%, respectively. Hydrogen bonds formed between Asn 142 and compound **17** (18.39%), but not with compounds **7** and **8**. Furthermore, the protease His164-compound **17** complex showed a lower occupancy of hydrogen bonds than the protease His 164-compound **7** and **8** complexes, with occupancy levels of 23.03% and 63.41%, respectively. These results suggest that the amino acid residues of the protease active site have fewer interactions with compounds **7** and **17** than with compound **8**. It may be probable that the strong interactions between the protease amino acid residues and compound **8** are crucial to the higher affinity binding and thermodynamic stability of the complex.

### 2.6. Principal Components Analysis (PCA)

**Figure 8:**
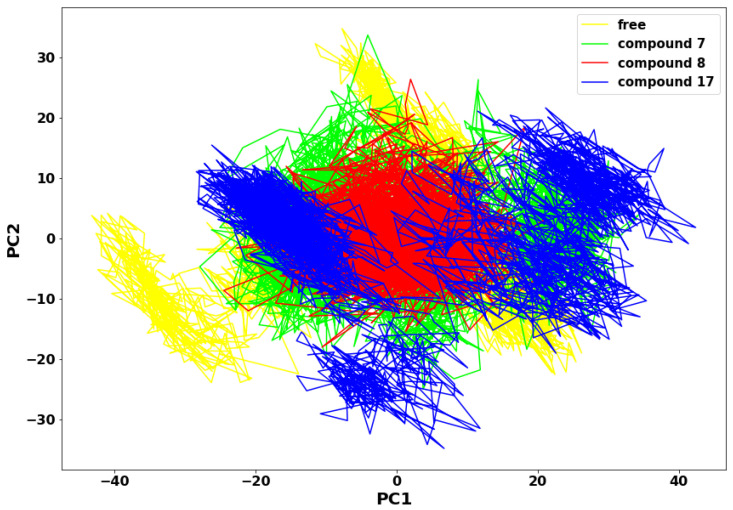
Principal Component Analysis plot.

The more compact the protein molecules, the more stable the protein complex. The motion of the molecules is also directly proportional to the compound’s molecular weight. In the principal components analysis (PCA) plot in Figure 8, compound **8** molecules appear less scattered and more compact when compared to compounds **7** and **17**. It is suggested that the substituted aromatic benzene ring contributed immensely to the thermodynamic stability of compound **8**. Therefore, the collective motions of SARS-CoV-2 Mpro-compound **8** are directly related to the protein stability and, consequently, its function. An overall collective motion can be characterised by a 2D projection of the trajectory plot in the important subspace of a system [25,26].

### 2.7. Per-Residue Energy Decomposition

**Figure 9:**
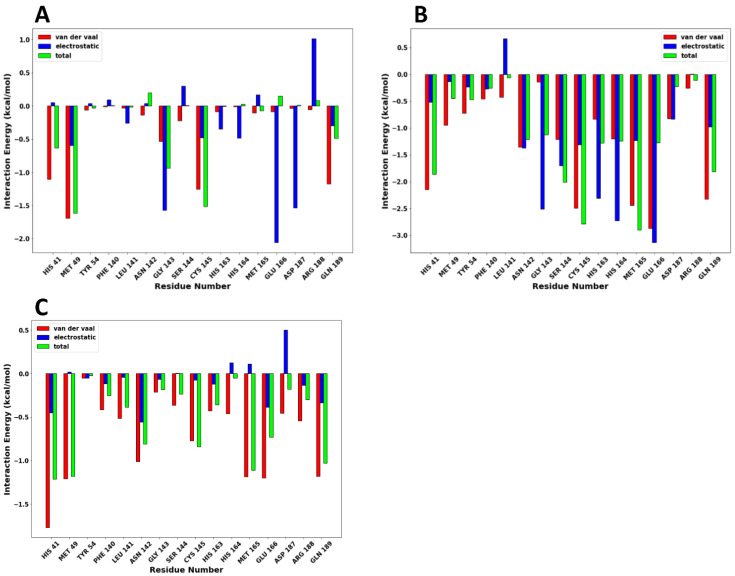
The energy decomposition plot of protein–ligand interactions per-residue are shown as follows: (**A**) compound **7**, (**B**) compound **8**, and (**C**) compound **17**, respectively.

The energy decomposition of protein–ligand interactions per-residue of SARS-CoV-2 Mpro with compounds **7**, **8,** and **17** complexes was calculated to unveil the amino acid residues essential for ligand-protein interactions. The energy decomposition of protein–ligand interactions per-residue is shown in Figure 9A (compound **7**), 9B (compound **8**) and 9C (compound **17**), respectively.

In compound **7**, residues Met49 (−1.615 kcal/mol), Cys145 (−1.515 kcal/mol), Gly143 (−0.937 kcal/mol), His41 (−0.635 kcal/mol), and Gln189 (−0.489 kcal/mol) made significant van der Waals energy contributions. Consequently, residues Glu166 (−2.060 kcal/mol), Gly143 (−1.575 kcal/mol), Asp187 (−1.533 kcal/mol), Met49 (−0.593 kal/mol), His164 (−0.486 kcal/mol), and Cys145 (−0.475n kcal/mol) exhibited electrostatic energy contributions, respectively. In compound **8**, residues Glu166 (−3.138 kcal/mol), His164 (−2.272 kcal/mol), Gly143 (−2.518 kcal/mol), His163 (−2.312 kcal/mol), Ser144 (−1.702 kcal/mol), Asn142 (−1.370 kcal/mol), Cys145 (−1.309 kcal/mol), Met165 (−1.234 kcal/mol), showed strong electrostatic energy contributions. Also, residues Glu166 (−2.874 kcal/mol), Cys145 (−2.495 kcal/mol), Met165 (−2.441 kcal/mol), Gln189 (−2.327 kcal/mol), His41 (2.150 kcal/mol) made significant van der Waal energy contribution. The high electrostatic and van der Waal energy contributions made compound **8** bind strongly with SARS-CoV-2 Mpro at the active site. The strong van der Waal energy forces from the catalytic dyad of the SARS-CoV-2 Mpro also contributed significantly to the binding of the drugs at the active site. Finally, compound **17** has a few strong residue contributions resulting in the low total binding free energy with the SARS-CoV-2 Mpro. This low binding free energy by compound **17** was mainly van der Waal energy contributed by residues His41 (−1.774 kcal/mol), Met165 (−1.189 kcal/mol), Gln189 (−1.182 kcal/mol), and Met49 (−1.210 kcal/mol).

## 3. Materials and Methods

### 3.1. Systems Preparations

The crystal structure of SARS-CoV-2 in PDB format was obtained from the protein data bank with PDB:1D 6LU7. The receptor was prepared using discovery studio [27] and Chimera software (ChemDraw 8 Ultra) [28]. The two-dimensional structures of the compounds were drawn using ChemDraw [29] and optimised to 3D with Avogadro [30]. The hydrogen atoms were added, and the compounds’ partial charges were assigned using the AM1-BCC [31]. The general amber force field (GAFF) assigned the atom types, bond orders, and van der Waals parameters. The Autodock Vina module [32] in chimera was used to dock the three selected compounds for this study into the SARS-CoV-2 main protease. The docked poses were selected based on visual inspection relative to the binding energy score within the acceptable RMSD value of <2 angstroms.

### 3.2. MD Simulations

The assisted model building and energy refinement 18 (Amber 18) [33] graphic processing unit (GPU) of PMEMD was employed to carry out 250 ns MD simulations for apo SARS-CoV-2 Mpro and SARS-CoV-2 Mpro in complex with compounds **7**, **8**, and **17**. The Amber force field FF14SB was used to parametrise the protein [34]. The LEAP module implemented in Amber18 was used to add hydrogen atoms to the receptor and counter-ion to neutralise the system [35]. The system was solvated using the TIP3P water box with a cut-off of 8 Å to the solute. The system utilised periodic boundary conditions, whereas the long-range electrostatics were handled using PMEMD in Amber18 with a cut-off of 12 Å. The initial minimization was carried out utilizing the restrained potential of 500 kcal/mol/Å^2^ in 1000 steepest descent steps and 1000 conjugate gradient steps on the solute. Subsequently, a 1000 step conjugate gradient minimisation unrestrained was done for the entire system. The system was heated gradually from 0 to 300 Kelvin using NVT canonical ensemble and a harmonic restraint of 5 kcal/mol/Å^2^ for the solute atoms with a one picosecond random collision frequency. An unrestrained equilibration of the system using NPT ensemble at 1 bar and 300 K was performed. Subsequently, a production MD simulation run of 250 ns was performed with an isothermal isobaric (NPT) ensemble and a Berendsen Barostat [36]. The coordinates were saved at intervals after each stage, and the MD trajectories were analysed using CPPTRAJ and PTRAJ [37]. The results were visualised using chimera molecular modeling software (UCSF Chimera software package), and the Origin software (Origin Lab, Northampton, MA, USA) was used to plot the graphs and charts [38].

### 3.3. Root Mean Standard Deviation (RMSD)

Root Mean Square Deviation was applied to measure displaced atoms or groups of atoms in the specified molecular dynamic simulation run [39,40]. We analysed the root mean square deviation trajectory of the α-carbon of the protein’s backbone with the CPPTRAJ module using Equation (1). The standard deviation of the interatomic distance between α-carbon backbone atoms of two amino acids *v* and *w* at *n* points represents *v_i_* as α-carbon coordinates in *v* at the time *i*, and *w_i_* is the coordinates of α-carbon atom in *w* at the time I [41,42,43,44].

(1) $$RMSD\left(v,w\right)=\sqrt{\frac{1}{n}\sum_{i=1}^{n}||v_{i}-w_{i}}|{|}^{2}$$

### 3.4. The Radius of Gyration (RoG)

The radius of gyration involves the measurement of a body’s distance from the centre of mass of a body where the entire mass is concentrated without altering its moment of rotational inertia, which the whole mass could be concentrated without changing its moment of rotational inertia. The RoG is also the equilibrium conformation of a protein within a given trajectory in a given molecular dynamic simulation run. The RoG explains atoms’ root mean square deviation from a given enzyme molecule’s common centre of gravity [45]. The RoG was determined using the following equation:

(2) $$r^{2}g=\frac{\sum_{v=0}^{k}W_{v}{\left(r_{v}-r^{-}\right)}^{2}}{\sum_{v=1}^{k}w_{v}}\;\;$$

where *r_v_* denotes the position of the *v^th^* atom, and *r* is the centre mass atom *v*. The mean value is determined by taking RoG values over the number of frames in a given trajectory [3].

### 3.5. Principal Components Analysis

Principal Components Analysis (PCA) was applied in obtaining aggregate movement of the coordinates that represent the overall dynamics of each trajectory. The covariance matrix was diagonalised to yield a set of eigenvectors and eigenvalues [46]. The CPPTRAJ module of AMBER 18 was used to strip the water and ions from the 250 ns MD trajectories of SARS-CoV-2 main protease apo, SARS-CoV-2-compound **7**, SARS-CoV-2-compound **8**, and SARS-CoV-2-compound **17** complexes [41]. The different molecular dynamic simulation trajectories were computed to determine the covariance matrix (C-α atoms) between residues j and p [47]. In-house scripts were used to calculate the first two principal components (PC1 and PC2) and generate the covariance matrix. The first two principal components correspond to the first two Eigenvectors of the covariance matrix. The PCA scatter plots were then constructed with Matplotlib [48,49].

### 3.6. Thermodynamic Analysis

The binding free energy analysis computes the endpoint energy landscape and subsequently provides essential information on the receptor–ligand complex interactions. In an ideal spontaneous reaction with equilibrium states of constant pressure and temperature, the receptor–ligand complex occurs when the system’s change in Gibbs free energies (ΔG) is negative. Contingent upon the fact that the receptor–ligand association is relative to the magnitude of the—ΔG, therefore, it is suggested that the stability of any given receptor–ligand complex is controlled by ΔG [38,50]. Furthermore, ΔG is determined by the initial and final thermodynamic states, irrespective of the pathway connecting the two states. The binding free energies of SARS-CoV-2-compound **7**, SARS-CoV-2-compound **8**, and SARS-CoV-2-compound **17** complexes were determined using the molecular mechanics/generalized-born surface area (MM/GBSA) method. The following equations, therefore, summarise the binding free energy:

ΔG_bind_ = G_complex_ − G_receptor_ − G_ligand_ (3)

ΔG_bind_ = E_gas_ + G_sol_ − TΔS (4)

E_gas_ = E_int_ + E_vdW_ + E_ele_ (5)

G_sol_ = G_GB_ + G_SA_ (6)

G_SA_ = γSASA (7)

where E_gas_ is the gas phase energy; E_int_ is the internal energy; E_ele_ the is the electrostatic (Coulomb) energy, and Evdw is the van der Waals energy. The gas-phase energy is estimated directly from the FF14SB force field terms. The solvation-free energy is decomposed into polar and non-polar states. The polar salvation G_GB_, contribution is evaluated by solving the GB equation. In contrast, the non-polar solvation contribution, G_SA_, is determined from the solvent-accessible surface area (SASA) was estimated using a water probe radius of 1.4 angstroms. T represents the temperature, and S is the total solute entropy. The individual amino acid contributions to the total binding free energy of the three complexes were calculated by the interaction energy decomposition analysis per residue using the Amber18 molecular mechanics/generalised-born surface area binding free energy method. We decomposed and analysed each contributing residue’s energy contributions interacting with the ligand at the protein’s active site. This total energy decomposition reveals the binding modes of compounds **7**, **8**, and **17** with the SARS-CoV2 main protease [4,5].

## 4. Conclusions

SARS-CoV-2 Mpro is an essential target for drug design and has been extensively studied. We theoretically investigated the previously reported compounds against SARS-CoV-2 Mpro to unveil insight observed in experiments. The selected compounds’ pharmacokinetics profile, physiochemical, and drug-likeness were assessed using the SwissADME web server. Compound **17** had a molecular weight of 307 g/mol, less than five rotatable hydrogen bonds, and less than 5 H-bond acceptors, and donors exhibited good blood–brain barrier permeation potentials. The docked compounds **7**, **8** and **17** showed the docking scores −6.56, −5.71, and −2.68 kcal/mol, respectively, suggesting that compound **7** has a better interaction with the binding site when compared with compounds **8** and **17**. Given the structural similarity of compounds **7** and **8**, both docking scores are closer (−6.56 and −5.71 kcal/mol) than the distant compound **17**, with the least stable interaction based on the docking score. The MD trajectories from the 250 ns MD production run were analysed to provide molecular insight into the dynamic behaviour of the systems upon ligand binding. However, the binding free energies assessed by the MM-GBSA indicate that compound **8** was the best binder. The substitution of isopropane with an aromatic benzene moiety is suggested to be responsible for the better binding potential of compound **8**, which exhibited the highest binding free energy of −49.37 ± 0.15 kcal/mol, followed by compound **7** (−39.83 ± 0.19 kcal/mol), whereas compound **17** showed the lowest binding free energy (−23.54 ± 0.19 kcal/mol). Additional intermolecular evaluations showed compound **8** possesses better binding potentials and suggested better drug candidates for inhibiting SARS-CoV-2 Mpro. The substitution of isopropane in compound **7** with an aromatic benzene ring in compound **8** significantly enhanced the drug’s ability to bind better at the active site of the SARS-CoV-2 Mpro. Finally, compounds **7** and **8** appear to bind better at the active site of SARS-CoV-2 Mpro when compared to compound **17**. However, these results are computational predictions and can further be confirmed in vivo through preclinical studies in SARS-CoV2 infection models. Therefore, we recommend a collaboration of computational chemists with experimentalists, as this promises to produce more enhanced and accurate predictions.

## Figures and Tables

**Table 1:** MMGBSA binding free energy contributions for SARS-CoV-2 Mpro in complex with compounds **7**, **8**, and **17**.

Complexes	ΔG_vdw_	ΔE_ele_	ΔE_bind_	ΔE_gas_	ΔG_sol_	ΔG_pol_	ΔG_nonpol_
Compound **7**	−45.47 ± 0.18	−36.17 ± 0.26	−39.83 ± 0.19	−81.63 ± 0.37	41.80 ± 0.18	47.70 ± 0.19	−5.89 ± 0.02
Compound **8**	−55.57 ± 0.14	−40.09 ± 0.27	−49.37 ± 0.15	−95.67 ± 0.31	46.30 ± 0.23	53.32 ± 0.23	−7.02 ± 0.02
Compound **17**	−30.93 ± 0.24	−4.54 ± 0.09	−23.54 ± 0.19	−35.48 ± 0.24	11.92 ± 0.09	15.56 ± 0.10	−3.64 ± 0.03

**Table 2:** Swiss ADME Pharmacokinetic and physiochemical profile of compounds **7**, **8** and **17**.

Compounds	Molecular Weight (g/mol)	No. of Rotatable H-Bond	No. of H-Bond Acceptor	No. of H-Bond Donor	CYP1A2 Inhibitor	CYP2C19 Inhibitor	CYPC2C9 Inhibitor
Compound **7**	587.71	21	7	4	No	No	No
Compound **8**	621.73	22	7	4	No	No	No
Compound **17**	307.13	3	3	1	Yes	Yes	Yes

**Table 3:** The hydrogen bond contributions of the compounds.

Complexes	Acceptor	DonorH	Donor	Percentage Occupancy	Average Distance
Compound **7**	GLY143@O	COMP7@H	COMP7@N2	28.09	2.87
	GLU166@O	COMP7@H2	COMP7@N4	25.14	2.86
	HIS164@O	COMP7@H	COMP7@N2	23.03	2.87
	CYS145@O	COMP7@H1	COMP7@N2	15.85	2.91
	HIS41@HD1	COMP7@H	COMP7@N2	10.78	2.92
Compound **8**	HIS164@O	COMP8@H2	COMP8@N4	63.41	2.86
	COMP8@O3	GLY143@H	GLY143@N	46.96	2.81
	GLU166@O	COMP8@H	COMP8@N2	10.38	2.87
	COMP8@O3	CYS145@H	CYS145@N	8.27	2.92
Compound **17**	COMP17@O	GLU166@H	GLU166@N	15.85	2.89
	HIS164@O	COMP17@H	COMP17@N	5.17	2.82
	COMP17@N1	GLY143@H	GLY143@N	3.64	2.92
	COMP17@N1	ASN142@HD22	ASN142@ND2	1.95	2.92

## Data Availability

The data presented in this study are available on request from the corresponding author.
